# Therapeutic Argentine Tango Dancing for People with Mild Parkinson’s Disease: A Feasibility Study

**DOI:** 10.3389/fneur.2015.00122

**Published:** 2015-05-27

**Authors:** Laura M. Blandy, Winifred A. Beevers, Kerry Fitzmaurice, Meg E. Morris

**Affiliations:** ^1^College of Science, Health and Engineering, School of Allied Health, La Trobe University, Bundoora, VIC, Australia

**Keywords:** Parkinson’s disease, tango, feasibility, depression, quality of life

## Abstract

**Background:**

Individuals living with Parkinson’s disease (PD) can experience a range of movement disorders that affect mobility and balance and increase the risk of falls. Low health-related quality of life, depression, and anxiety are more common in people with PD than age-matched comparisons. Therapeutic dance is a form of physical activity believed to facilitate movement and therapy uptake. As well as being enjoyable, dancing is thought to improve mobility, balance, and well-being in some people living with PD. The primary objective of this study was to evaluate the feasibility and safety of a 4-week Argentine tango dance program for people with PD.

**Methods:**

Six community dwelling individuals with mild to moderate PD were recruited from Parkinson’s support groups, movement disorder clinics, and the PD association in Australia. To minimize falls risk, participants were required to be <75 years of age and physically independent (Hoehn and Yahr stages I–III). They were also required to speak English. Participants attended a 1-hour dance class at a dance studio twice per week for 4 weeks. A professional dance instructor led and choreographed the Argentine tango dance classes. Physiotherapists were present to assist participants during the class and served as dance partners as necessary. The primary outcome was feasibility, which was determined by measures of recruitment, adherence, attrition, safety (falls, near misses and adverse events), and resource requirements. Secondary measures included the Beck Depression Inventory and the Euroqol-5D, administered at baseline and post intervention. Therapy outcomes pre- and post-intervention were analyzed descriptively as medians and interquartile ranges and using Wilcoxon matched pair signed-rank tests.

**Results:**

The Argentine tango dance intervention was shown to be safe, with no adverse events. Adherence to the dance program was 89%. Depression scores improved after intervention (*p* = 0.04). Some challenges were associated with the need to quickly recruit participants and physiotherapists to act as dance partners during classes and to monitor participants.

**Conclusion:**

The 4-week, twice weekly Argentine tango dancing program was shown to be feasible and safe for people with mild-to-moderately severe PD.

## Introduction

Idiopathic Parkinson’s disease (PD) is a progressive neurological disorder associated with reduced mobility, falls, and reduced quality of life (QOL) (1). International guidelines endorse exercise therapy to retrain balance and preserve physical capacity for individuals living with PD (2). Meta-analyses have also shown exercise therapy to have positive effects with regards to physical functioning, balance, and health-related QOL (3, 4). Despite the potential benefits of movement rehabilitation, long-term adherence to traditional exercise programs can be problematic (5). A systematic review evaluating exercise adherence in PD reported that reduced motivation was a common reason for reduced participation (6). This demonstrates a need for community-based physical activity programs that facilitate uptake and enjoyment (7).

Emerging evidence suggests that therapeutic dance may be an appropriate and enjoyable form of physical activity for some individuals with PD (8). Dance may address some of the physical impairments in PD through teaching movement strategies, challenging balance, and improving physical fitness (9, 10). The musical rhythm could become an auditory cue to engage cortical control of movement, which in turn might potentially enhance motor learning (11). Preliminary trials suggest dance can facilitate improvements in gait, balance, and motor impairment in comparison to exercise (12), physiotherapy (13), and control conditions (8, 14–16). It has been proposed that therapeutic dance may also facilitate QOL and well-being through enabling movement expression and building social connections (17).

The Argentine Tango dance genre is arguably one of the most suitable dance forms for people with PD (8, 14, 15). It has been proposed to target the movement impairments of PD with strong musical rhythms that trigger movement and enable greater amounts of physical activity. Compared to other dance genres, the choreography can be designed to train specific movement strategies such as walking backwards and turning (18). Furthermore, as a partnered form of dance, tango may facilitate interpersonal connections that positively affect QOL and mood (18).

While a growing number of pilot studies have explored the effects of dance on movement disorders in PD, there is a paucity of feasibility data and recommendations that allow researchers to design future protocols (19). Comprehensive exploration of the safety of specific dance genres is still required given that individuals with PD have a propensity to fall (20). This research also focused on QOL and depression outcomes, as there is little published literature on the effects of therapeutic dance on perceived QOL and mood for adults with PD.

The primary purpose of the current study was to evaluate the feasibility and safety of an Argentine tango dance intervention and to provide recommendations for future research. The specific aims were to: (i) determine if 4 weeks of twice weekly Argentine tango dance classes were feasible and safe for people with PD, allowing the development of recommendations for a future research protocol and (ii) measure the within-group change for depressive symptoms and health-related quality of life (HRQOL) following participation in the dance classes.

## Materials and Methods

### Design

This feasibility study adopted a single-group, pre-/post-test design and received ethical approval by the La Trobe University Faculty of Health Sciences Human Ethics Committee (ref. FHEC13/026). The trial was registered with ANZCTR, number ACTRN12613001058763.

We recruited participants with idiopathic PD from the metropolitan Melbourne region in Australia. As the current body of literature does not provide an arbitrary method for determining sample sizes in pilot research (21), there were various considerations involved in determining sample size. Primarily, it was anticipated that a dance class with six to eight participants could be adequately monitored (18).

### Participants

The eligibility criteria tested what was planned for a future clinical trial. Criteria were also established to maximize safety and reduce risk of falls during the interventions. Eligibility criteria therefore included: (i) a diagnosis of idiopathic PD confirmed by a neurologist; (ii) mild-to-moderate disease severity (Hoehn and Yahr stages I–III) (22); (iii) community dwelling; (iv) aged 18–75 years; and (v) medically safe to participate. Volunteers were excluded if they were unable to provide informed consent (Mini Mental State Examination, MMSE, score <24) (23) and did not have sufficient English to be able to follow instructions. Volunteers were recruited through local Parkinson’s support groups, movement disorder clinics, and the website and newsletter of Parkinson’s Victoria, Australia.

At intake, participants signed informed consent and were screened for eligibility. The Unified Parkinson’s Disease Rating Scale motor subsection (MDS-UPDRS-III) (24) and a health status screening form to identify any comorbidities that could require extra monitoring during intervention were also administered. They were then asked to provide written consent to contact their medical practitioner for written medical clearance.

### Intervention

Participants were invited to participate in 1-hour dance classes that ran twice weekly over 4 weeks at a dance studio in metropolitan Melbourne. They were required to make their own way to classes, and were provided with taxi vouchers if required. Classes of Argentine tango choreography were designed and led by a professional dance instructor, with experienced physiotherapists present to spot participants at risk of falls and serve as dance partners. People who were partners were all able-bodied participants. Classes included regular rest breaks, typically at 20-min intervals, to minimize fatigue (18). Modifications were made to the intervention by physiotherapists to reduce falls risk as needed. The participants essentially had the same class, although as is customary with tango dancing, there were some gender differences in the dance routines. Women are usually the followers in tango, and were required to frequently do steps backwards, in contrast to men. All of the participants were using PD medications and they were in the “on” phase of the medication cycle during the dancing classes. An outline of class structure and content is detailed in Table 1.

**Table 1:** **Class structure and content**.

Section	Time (min)	Activity	Objectives
Warm up	0–10	Large amplitude movement of each joint through full range Rib/pelvic dissociation movements	Prepare the body/muscles for dance Increase range of motion at joints Decrease trunk rigidity
Individual step practice	10–25	Introduction to rhythm and beat of music Stepping forwards, backwards, sideways in patterns following dance instructor Seated practice footwork	Training ear to identify external cues to movement Practice movement coordinated to rhythm of music Practice weight shift, large amplitude steps, challenging balance Practice of complex foot and ankle movement patterns while seated
Break	25–30	Seated rest	Prevent fatigue
Partnered Practice tango steps	30–50	Partnered practice with modified tango “embrace” Shared leading and following roles Practice of stepping patterns in time with music Encouraging postural awareness with respect to position partnered “embrace” Practice turning, change of direction	Address motor difficulties with gait speed, step length, movement initiation coordination, weight shift Aerobic training Balance retraining Teaching movement strategies for complex movement such as turning/walking backwards
Break	50–55	Seated Rest	Prevent fatigue
Cool down	55–60	Breathing Gentle stretching Close with applause	Relaxation Encourage deep breathing with basal expansion Establish sense of accomplishment

### Primary outcomes

(i) Feasibility of Argentine tango dance was determined by quantifying recruitment rates, adherence, attrition, safety, and resource requirements. Feasibility outcomes were monitored throughout the recruitment phase and over the 4-week intervention phase, and data were entered into customized forms.
(ii) Recruitment: researchers collected data regarding the time taken to recruit, the number of respondents to advertisements, and proportions considered eligible or ineligible to participate.
(iii) Adherence and attrition: participants were required to sign an attendance form at each of the dance sessions. Reasons for non-attendance were recorded as (i) medical (specified as related or unrelated to the dance intervention), (ii) disinterest, (iii) personal, or (iv) difficulty accessing venue. The number who withdrew and reason for withdrawal were also recorded.
(iv) Safety: adverse events (fall, injury, or medical emergency), near misses (slips or trips) and complaints of pain, stiffness, or fatigue were documented. It was also noted if participants required “hands on” assistance for balance and if “on the spot” modifications were made to the intervention for safety purposes.
(v) Resource requirements: to document whether attaining the personnel required was feasible (19), the attendance of supervising physiotherapists was recorded.

To objectively establish if the proposed research protocol was successful, the following *a priori* criteria for feasibility success were developed (19): (i) recruitment strategy and screening process enable recruitment of eight participants within 1 month; (ii) adherence ≥70%, which has been deemed as high in older adults with a physical impairment (25); (iii) attrition ≤15%, an arbitrary figure for acceptable attrition established by the PEDro scale (26); and (iv) safety: the dance intervention was considered safe if there were no falls or injuries during the intervention and no “hands on” assistance was required for prolonged periods.

### Secondary outcomes

The effects of the intervention on HRQOL were measured with the Euroqol-5D (27, 28), a validated measure of HRQOL in PD, using the rating questionnaire and the visual analog scale (VAS). The summary index was computed for the descriptive system using normative data sets from the United Kingdom, with a possible value between −0.59 and 1, where 1 represents “full health.” The VAS scale is a self-perceived rating of health status scored from 0 to 100, with 100 indicating best imaginable health state. The Beck Depression Inventory (BDI) (29) was used to measure depression. This questionnaire has established validity and reliability for assessment of depressive symptoms in the PD population (30). The BDI scores are tallied to obtain a score out of 63, with a higher score indicating greater severity of symptoms. Both questionnaires were administered at baseline and at the end of the 4-week intervention phase.

### Statistical analysis

Therapy outcomes at pre- and post-intervention were analyzed descriptively as medians and interquartile ranges (IQR) to better describe the small data set and control for extremes in scores (31). Where participant data were missing at post-test, baseline data were carried over to complete the analysis. To establish statistical significance of within-group change between the two time points, a Wilcoxon matched pair signed-rank test was performed with *p*-value set at <0.05. A non-parametric technique was necessary given data were collected from a small sample and was not normally distributed (31). Statistical analysis of therapy outcomes was performed using SPSS statistics (IBM, Armonk, NY, USA).

## Results

### Participants

Participant demographic data are detailed in Table 2. Participants had mild PD as indicated by low mean scores on the UPDRS part III, motor examination, and with all participants scoring 2 on the modified Hoehn and Yahr scale.

**Table 2:** **Participant characteristics**.

Variable	Participants (*n* = 6)
Age (years)	64 (6.28)
Sex (male/female)	3/3
MMSE/30	28.6 (1.50)
HY (median, IQR)	2 (2–2)
MDS-UPDRS 3	20 (5.89)
Duration of PD (years)	8.57 (4.0)

*Values are means (SD) unless stated otherwise. MMSE, mini-mental state examination; HY, modified Hoehn and Yahr scale; IQR, inter quartile range; MDS-UPDRS, Movement Disorders Society-Unified Parkinson’s Disease Rating Scale; PD, Parkinson’s disease*.

### Feasibility

#### Recruitment

**Figure 1:**
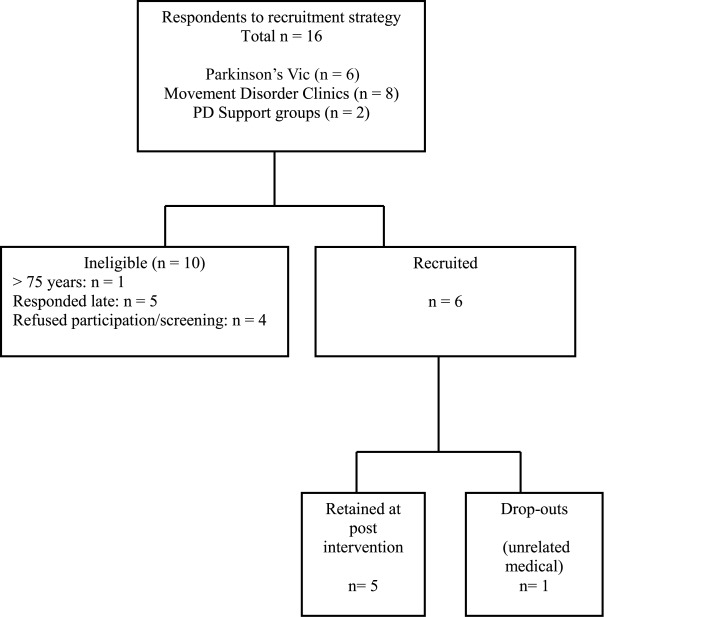
**Flow of participants through recruitment and intervention phases**. Parkinson’s Vic, Parkinson’s Victoria; PD, Parkinson’s disease; *n*, number.

The proposed recruitment strategy did not meet *a priori* criteria. Six individuals were recruited over 2 months. Figure 1 indicates the flow of participants in the recruitment and intervention phases.

#### Adherence

Attendance to the dance classes was 89%, exceeding the *a priori* criterion of 70%. One participant was unable to attend one class due to an unrelated medical matter, and another participant was unable to attend two classes secondary to a scheduled vacation.

#### Attrition

One participant dropped out for the final week of the dance intervention (including the post-intervention assessment) citing a combination of medical and personal reasons. Attrition rate was thus 17% for the 4-week dance intervention, failing to meet the feasibility criteria of 15%.

#### Personnel Requirements

Attendance of the supervising physiotherapists throughout the program was 86%, lower than the required 100% to provide dance partners for participants.

#### Safety

There were no falls, injuries, or medical emergencies throughout the intervention period. Three participants withdrew from the class on separate occasions due to (i) one episode of transient vertigo following rehearsal of fast-paced turning; (ii) pain at an arthritic hip joint; and (iii) medication-related motor complications. There was one documented “near miss” without injury where a participant destabilized secondary to a dyskinetic tick. Incidents of mild pain at the back (*n* = 1), shoulder (*n* = 1), foot/ankle (*n* = 1), and hip (*n* = 1) were also documented. In health status screening forms, these participants cited arthritis at these joints. “Hands on” assistance was required in only one instance and distant (1 m) supervision was provided to all participants at all other times.

#### Modifications to Dance Intervention

The intervention was modified on four occasions to ensure participant safety; no walking backwards in the first session, steps requiring single leg stance to be performed while seated, practice of turns to be performed over an arc rather than on the spot, cessation of dance to music with fast tempo. The tango hold position was also modified for one participant due to shoulder pain.

### Health-related quality of life and depression

The EuroQol median score increased from a baseline score of 0.796 (IQR: 0.73–1.00) to a post intervention score of 0.890 (IQR: 0.80–1.00), indicating improvement; however, this did not reach statistical significance (*p* = 0.317). There was a small but not significant improvement in the VAS score from a median score of 80 (IQR: 77.5–91.25) at baseline to 82.5 (IQR: 77.5–91.25) post intervention (*p* = 0.854).

Beck Depression Inventory scores at both time frames were distributed around the lower end of the scale, suggesting that overall depressive symptoms were low (32). The BDI scores decreased, indicating an improvement in depression, from a median score at baseline of 5.5 (IQR: 4.75–8.50) to a median of 0 (IQR: 0–5.50) post intervention, which reached significance (*p* = 0.042).

## Discussion

The Argentine tango dance intervention was found to be feasible for this sample of people with comparatively mild PD. It was shown to be safe with no serious adverse events, such as falls, injuries, or medical emergencies, occurring. The minor adverse events were mostly related to comorbidities, and were readily managed with modifications to dance routine and steps. Modifications were, however, made infrequently. All documented modifications reflected the recommendations for tango classes for adults with PD developed by Hackney and Earhart (18), and Rios Romenets, Anang (16). It is evident that the safety of the intervention was enabled by the presence of supervisors to monitor instability and to advise appropriate modifications. Formal education of dance instructors on screening and monitoring for adverse responses to interventions may result in earlier modifications to the interventions and a subsequent minimization of adverse outcomes (33). The selected dosage was shown to be safe and appropriate, and none of the participants reported pain, stiffness, or fatigue after classes.

Recruitment in the short time frame available was challenging. Researchers continued to receive expressions of interest in the month following commencement of the dance program, indicating a 3-month recruitment period may have successfully recruited eight participants. Recruitment difficulties are common in PD research (34), with similar issues reported by Batson (33), who rescheduled her dance program secondary to recruitment difficulties, and Duncan and Earhart (14), who reported only 50% of participants screened were eligible to participate in a tango dance intervention. It is possible that the potential benefits of dance are not widely understood, narrowing those who volunteer to participate (33). People with Parkinson’s were more reluctant to participate if they had to commute long distances. This is supported by research identifying transportation as a barrier to exercise uptake for individuals with movement disorders (35).

Adherence to the Argentine tango dance program was high, satisfying feasibility criteria (25). Adherence has only been reported in a small number of studies evaluating dance therapy for PD. Duncan and Earhart (14) reported 78% adherence over 12 months of tango, Volpe et al. (13) showed 90% adherence with 6 months of Irish dance, and 90% adherence over 3 weeks of modern dance was reported by Batson (33). The high adherence to dance therapy supports the hypothesis that therapeutic dance may facilitate uptake and enjoyment (9). However, these findings may have been influenced by selection bias, where individuals that volunteer to participate are more likely to adhere to a program (36). Further research is warranted to explore adherence to a longer program, and to determine if systematic differences in adherence exist between tango and a second dance genre, or a comparison intervention. Attrition was relatively low over the short intervention phase. While this narrowly fell short of *a priori* criteria, a 100% retention rate was necessary to meet this criterion as the eventual sample included only six participants. Importantly, data showed that the participant lost to post-intervention assessment did not report dislike or disinterest in the intervention, citing personal reasons for discontinuing.

This study adds to the limited body of evidence evaluating the influence of dance therapy on outcomes related to QOL and well-being, which are important to a holistic management of individuals with PD (28). This pilot study identified significant improvements in depressive symptoms and a trend toward improved HRQOL. For BDI scores, this was not only statistically significant but also research indicates a change from 5.5 to 0 may also be clinically meaningful (32). To the authors’ knowledge, this is the first study that has found positive effects of dance for depressive symptoms in adults with PD. The HRQOL findings support the work of Hackney and Earhart (37) and Volpe and colleagues (13), who found small, non-significant improvements in HRQOL compared to control and physiotherapy. Like this feasibility study, it is likely these preliminary trials were underpowered and may have failed to identify significant effects.

There are a number of factors that could have contributed to these observed improvements in depression and HRQOL. As a rehabilitative therapy, tango may improve specific functional impairments (18) that are known to influence perceived QOL for adults with PD (38). Research shows exercise to be an effective treatment for depression (39), and Ballroom dance has been shown to improve depression in the geriatric population (17). It is thought that dance may also improve well-being through enabling emotional expression and engagement (17), as well as building social support networks which may improve QOL for adults with PD (9).

There are a number of limitations of this pilot study. The small size of the sample decreases the external validity of findings (19, 40). Similarly, the short duration of the intervention phase means that few inferences can be made regarding the longer-term adherence and retention in adults with PD. Without a pilot control group, it cannot be determined if a comparison intervention (e.g., physiotherapy, exercise, or another dance genre) is also feasible pertaining to *a priori* criteria or if there are systematic differences between two intervention groups. Without a control group, this study could not counter for other potential factors that may have contributed to the observed improvements in therapy outcomes such as natural recovery or regression to the mean. Data were collected on a sample that was homogenous in disease severity; thus, conclusions regarding the safety of the dance intervention may not be applicable to individuals assessed as Hoehn and Yahr stages III and higher where postural instability is a marked impairment (22). This study did not evaluate participant acceptability of the intervention, which is suggested to be an important variable in pilot research (19). Furthermore, this study did not monitor if participants were concurrently undergoing active therapy for depression, which could have contributed to the observed improvement.

To facilitate the safety of the intervention delivery, it is proposed that dance instructors are educated regarding the disease processes, movement impairments, and likely comorbidities experienced by adults living with PD. Practical sessions would be useful to train instructors in pacing and movement selection (33). These sessions could educate instructors in therapeutic cuing, which could maximize movement and enhance motor learning for participants. Additional literature regarding community-based exercise programs for PD advocates basic training in first aid, emergency procedures, and exercise physiology (7).

It is recommended that the next stage of pilot research adopts a recruitment strategy based on the projection of recruiting 8–10 eligible participants over a 3-month period. Additionally, greater consideration should be given to location of the dance studio to limit barriers associated with long travel.

It is necessary for participant safety that the appropriate supervisors are present to monitor for fatigue, instability, and to provide appropriate suggestions regarding modifications. Hackney and Earhart (18) suggest that physiotherapy students may provide enthusiastic assistants, with the appropriate knowledge in risk management.

## Conclusion

A 4-week Argentine tango program was safe and enjoyable for people with relatively mild Parkinson’s and was associated with alleviation of depression in some. There remains a need to verify the safety of the intervention for people with PD, who are more disabled or with a greater fall risk. Arguably, people with more advanced disease and with a greater fall risk might show greater gains with this type of physical activity.

## Author Contributions

LB, MM, and WB participated in the design of the study, oversaw data collection, data analysis, preparation of the manuscript, gave final approval of the version to be published, and agreed to be accountable for all aspects of the work.

## Conflict of Interest Statement

The authors declare that the research was conducted in the absence of any commercial or financial relationships that could be construed as a potential conflict of interest.
